# Identification and preliminary study of a novel interacting protein SCRIB with fibroblast activation protein in OSCC: SCRIB interacts with fibroblast activation protein in OSCC

**DOI:** 10.3724/abbs.2023102

**Published:** 2023-06-19

**Authors:** Shuyan Liu, Pu Wang, Lingyun Ye, Chanjuan Liu, Wei Xiao, Chenyang Gao, Xinyi Huang, Jinxing Gao

**Affiliations:** 1 Center for Plastic & Reconstructive Surgery Cancer Center Department of Dental Medicine Zhejiang Provincial People’s Hospital (Affiliated People’s Hospital Hangzhou Medical College) Hangzhou 310014 China; 2 College of Animal Science and Technology Zhejiang A&F University Hangzhou 311300 China

Oral squamous cell carcinoma (OSCC) is a common oral malignant tumor. It can occur in the skin and mucosa, mostly in the tongue, palate, and gingiva. Lymph node metastasis usually occurs, and distal metastasis may also occur in the late stage [1]. Currently, surgery is the main treatment method, combined with radiotherapy and chemotherapy, and once the timing of cancer treatment is delayed, the patient’s speech, eating and survival will be seriously threatened. Thus, exploring the pathway of OSCC and finding new thermal targets are basic problems to be solved [2].

The occurrence of OSCC is the result of the interaction of multiple genes, involving the activation of oncogenes and the dysfunction of tumor suppressor genes, abnormalities in the cell energy metabolism signal pathway and cell differentiation signal pathway, and changes in the extracellular environment promoting tumor cell invasion, metastasis and immune escape [3]. Proteins play a key role in the process of carcinogenesis and cancer development [4]. In recent years, the development of proteomics and mass spectrometry (MS) has been helpful in studying the role of proteins in tumours [5]. Research on the interaction protein network in OSCC is needed to elucidate the regulatory mechanism of the occurrence and development of OSCC, which is helpful for the diagnosis and treatment of OSCC [6].

FAP is known to be overexpressed in breast, colorectal, pancreatic, lung, bladder, ovarian and other cancers. In these cancers, FAP is usually heavily expressed in the stroma and has thus become a universal marker of cancer-associated fibroblasts (CAFs). *FAP* knockdown in SKOV3 ovarian cancer cell lines results in decreased FAP expression in surrounding fibroblasts and decreased tumor growth, volume and proliferation [7]. In oral squamous cell carcinoma, knockdown of *FAP* results in decreased growth and metastasis *in vitro* and *in vivo*. Silencing of *FAP* reduces the activation of pRb and oncogenic cell cycle regulators, including CCNE1, E2F1, and c-Myc, but elevates the expressions of tumor suppressors such as p27 and p21. Furthermore, *FAP* silencing significantly decreases the expressions of phosphorylated PI3K, AKT, MEK1/2, ERK1/2, and GSK3b, whereas their total levels remain unchanged. A previous study confirmed that fibroblast activator protein level is significantly upregulated in OSCC compared with that in the control oral mucosa, suggesting that fibroblast activator protein participates in the development of OSCC. Further experiments confirmed that *FAP* knockdown in OSCC significantly reduces the growth of OSCC cells [8]. The regulatory mechanism of FAP-interacting proteins in OSCC is unclear, and further research is needed.

To clarify that the protein-protein interaction is important during the occurrence and development of OSCC, the associated protein from OSCC cells was screened by coimmunoprecipitation (co-IP) using FAP as the bait. In our previous research, the associated scribble planar cell polarity protein (SCRIB) of FAP in OSCC cells was obtained by IP and mass spectrometry (MS). To identify it, the interaction between SCRIB and FAP was confirmed by yeast two-hybrid assay. Amplification of FAP-specific DNA segments was performed using forward primer 5′- cgcGGATCCAGAGTTCATAACTCTGAAGAAAATACAAT-3′ and reverse primer 5′- ccgCTCGAGGTCTGACAAAGAGAAACACTGCTTTAG-3′ by polymerase chain reaction (PCR) from OSCC and then constructed on the pGBKT7 vector as the bait. The pGBKT7-FAP plasmid-transformed Y2HGold cells were plated on SD/–Trp/X plates for self-activation activity detection, and Y2HGold cells containing the pGADT7-SCRIB plasmid were plated on SD/–Leu/X-α-Gal plates. The results showed that the FAP and SCRIB proteins have no autoactivation activity in yeast ( Figure 1A).

**Figure 1:**
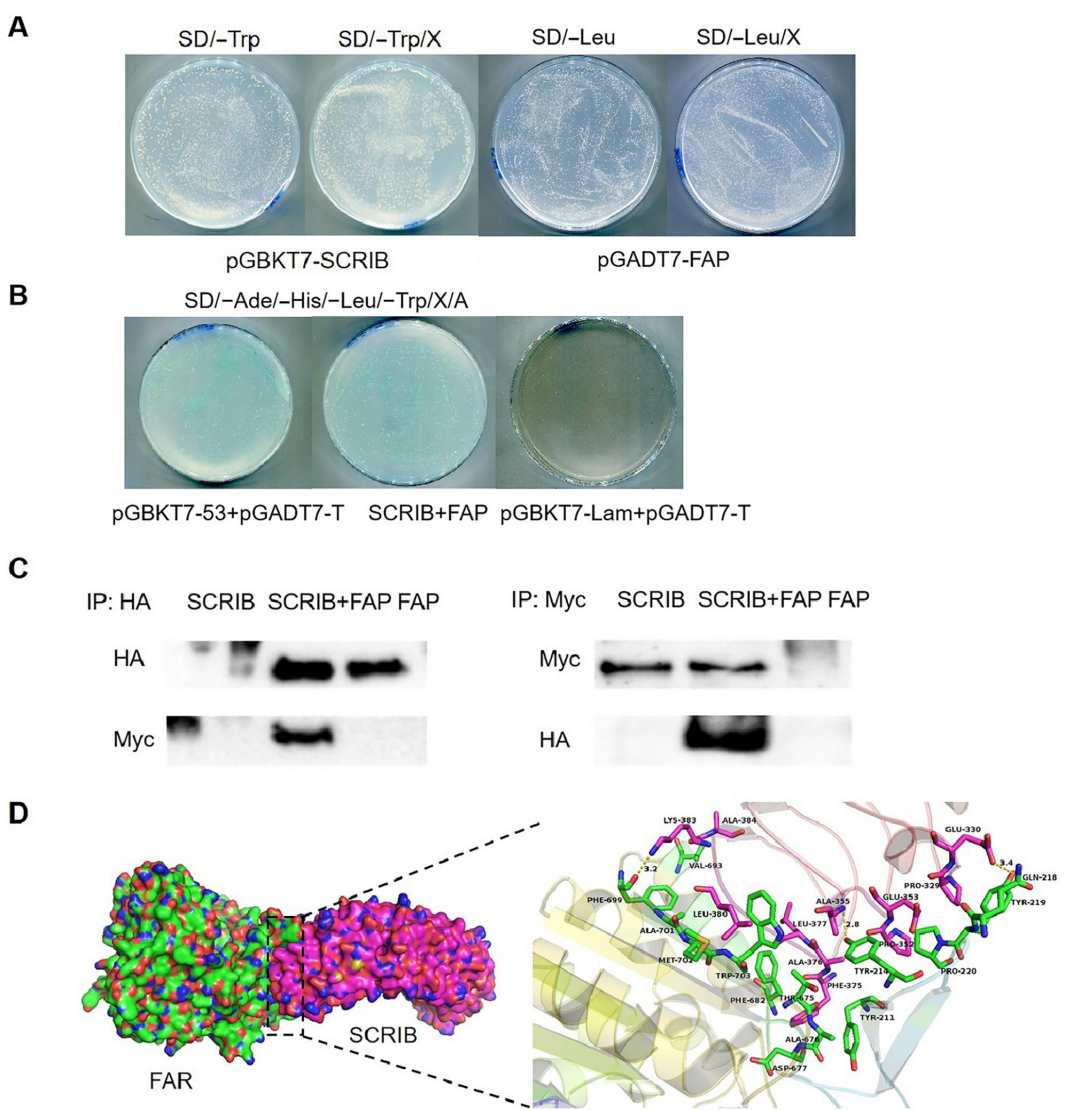
Figure 1 Identification of the FAP-SCRIB protein interaction by co-IP and yeast two-hybrid assay Detection of autoactivation of pGBKT7-FAP and pGADT7-SCRIB. Y187 strains were transformed with pGBKT7-FAP, and the transformant was grown on SD/–Trp/X-α-Gal. Similarly, the transformant with pGADT7-SCRIB was grown on SD/–Leu/X-α-Gal. (B) α-Galactosidase activity of the transformant with pGBKT7-FAP+pGADT7-SCRIB was detected. Transformant with pGBKT7-53+pGADT7-T was used as a positive control, and pGBKT7-Lam+pGADT7-T was used as a negative control. (C) The interaction of FAP with SCRIB was analysed by co-IP using anti-HA antibodies and anti-Myc antibodies. (D) Detailed view of the binding site between FAP and SCRIB.

Next, the interaction was demonstrated by yeast two-hybrid screen using the bait containing the pGBKT7-FAP plasmid and the prey containing the pGADT7-SCRIB plasmid on SD/‒His/‒Ade/ –Trp/–Leu/X/A plates. The results showed that SCRIB and FAP still showed a positive interaction ( Figure 1B). The sequencing results revealed that the insert containing the cDNA of scribble planar cell polarity protein has 100% sequence identity with SCRIB (NM_015356.5, https://www.ncbi.nlm.nih.gov/nuccore/). The potential biological functions of SCRIB (Q14160, https://www.ncbi.nlm.nih.gov/protein/) were predicted to be related to cell migration; cell proliferation; and protein localization to adherens junction. The interaction between FAP and SCRIB was further analysed by coimmunoprecipitation (co-IP). The recombinant plasmids pCMV-3×flag-FAP (296‒2302 bp) and pCMV-HA-SCRIB (510‒2855 bp), pCMV-3×flag-FAP only, or pCMV-HA-SCRIB only were transfected separately into 293T cells using liposomal reagent (Invitrogen, Carlsbad, USA). The extracts were harvested 24 h after transfection and then precleared with protein A/G beads. Subsequently, co-IP was performed using an anti-HA antibody followed by western blot analysis with an anti-Myc antibody. SCRIB was found to interact with FAP but not with the control ( Figure 1C). The binding of FAP (green) with SCRIB (pink) is shown in Figure 1D. The binding mode between the FAP and SCRIB proteins was analysed by molecular docking methods with the ZDOCK server. The ZDOCK server was used for docking amino acid molecules. PyMoL 2.4 software was used to plot and analyse the top scored docking results ( http://www.pymol.org/). Hydrophobic interaction was observed between Vla693 of FAP and Ala384 of SCRIB, Met702 of FAP and Leu380 of SCRIB. Furthermore, Tyr211 of FAP formed CH-π interactions with Phe375 of SCRIB. Pro220 and Tyr214 of FAP interact with Pro352 of SCRIB by π-π formation. Importantly, the interactions between the Tyr219 of FAP and Glu330 of SCRIB (bond length: 3.4 Å), Tyr214 of FAP and Ala355 of SCRIB (bond length: 2.8 Å), and Phe699 of FAP and Lys383 of SCRIB (bond length: 3.2 Å) are the main binding sites by hydrogen bonds between FAP and SCRIB. Molecular mimicking provides us with the predicted interaction regions between FAP and SCRIB and reasonable information for further research on the binding sites between FAP and SCRIB. These results confirm the interaction between SCRIB and FAP, which has not been reported. In various mammalian systems, SCRIB regulates the PI3K-AKT pathway. First, SCRIB negatively regulates AKT activity by binding to the protein phosphatase PHLPP (pleckstrin homology domain and LRR protein phosphatase) and anchoring it to the plasma membrane [9]. In the regulation of AKT, SCRIB forms a tripartite complex with PHLPP and AKT, thereby inhibiting AKT activity. However, when SCRIB expression is downregulated, PHLPP is released and AKT activity is increased, resulting in increased cellular growth, proliferation, and survival.

Small interfering RNA (siRNA) can be used to specifically silence abnormally expressed genes in tumor cells to understand the effects of these genes on tumor cell proliferation, differentiation, apoptosis and other biological functions. At present, siRNAs have become the research focus of tumor molecular targeted therapy. SCRIB is a tumor suppressor and may play a significant role in the process of cell migration, proliferation and invasion in the occurrence and development of cancer. SCRIB siRNAs and NC-siRNA ( Supplementary Table S1) were synthesized by OBiO (Shanghai, China) and transfected into SCC-9 cells using high-efficiency interfering RNA transfection reagent (Invitrogen). NC-siRNA-transfected SCC-9 cells served as the negative control group. TUNEL is a common method to detect broken DNA fragments produced by the apoptosis signal cascade. Red fluorescence was observed in apoptotic cells ( Figure 2A). The protein level of SCRIB in the siRNA group was less than that in the control group. TUNEL results showed that the number of positive cells in the si-SCRIB group was significantly less than that in the si-NC group ( Figure 2B).

**Figure 2:**
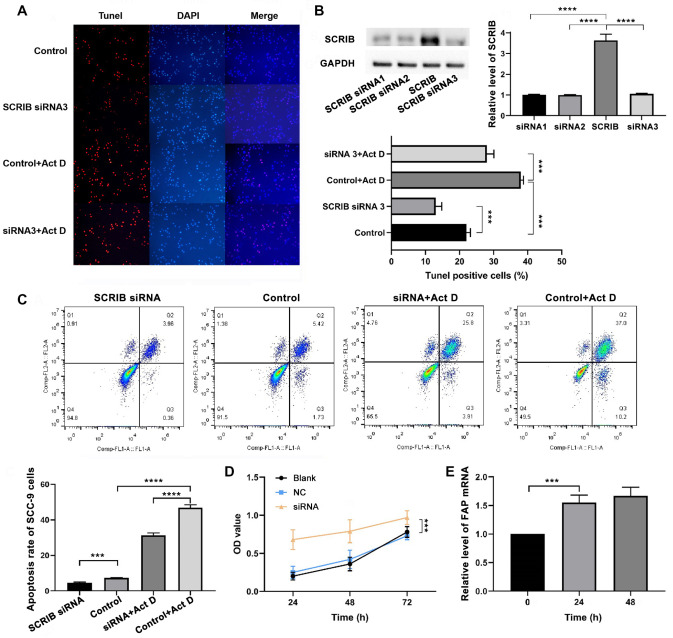
Figure 2 *SCRIB* knockdown inhibits the apoptosis and promotes the proliferation of SCC9 cells by negatively regulating FAP expression level (A) Apoptotic SCC-9 cells were detected by TUNEL assay under a fluorescence microscope in the control, SCRIB siRNA, control+Act D, and SCRIB siRNA+Act D groups. (B) Western blot analysis results showing SCRIB expression in the SCRIB siRNA 1, siRNA 2, siRNA 3 and si-NC groups at 72 h after transfection. The percentage of TUNEL-positive cells in the SCRIB siRNA, control, control+Act D and SCRIB siRNA+Act D groups ( n=3). (C) The percentage of apoptotic cells after SCRIB knockdown ( n=3). (D) A CCK-8 assay was used to detect the growth rates of SCC9 cells after SCRIB knockdown. (E) After SCRIB knockdown, the FAP mRNA level was analysed by RT-PCR at 24 h and 72 h. Data are shown as the mean± standard deviation (SD). *** P<0.001, **** P<0.0001 versus the control group.

The Annexin V/PI staining was used to detect the apoptosis/necrosis rate of SCC-9 cells after *SCRIB* knockdown. The apoptotic rate of SCC-9 cells in the si-SCRIB group was significantly lower than that in the control group and the si-SCRIB+Act D group at 24 h after downregulation of SCRIB expression ( Figure 2C). Depleted SCRIB promoted cell growth, as determined by CCK-8 assay ( Figure 2D). The expression of FAP was significantly increased 24 h and 48 h after *SCRIB* knockdown ( Figure 2E). Taken together, these results suggest that SCRIB attenuates cell growth by negatively regulating FAP expression *in vitro*. To identify the effect of SCRIB on the cell growth and apoptosis of SCC-9 cells, the levels of p-P13K, p-Akt, cleaved Caspase3 and FAP protein for 48 h were detected by western blot analysis after *SCRIB* knockdown. The results showed that the levels of p-P13K and p-Akt were increased significantly, the level of cleaved Caspase3 was decreased significantly, and the level of FAP protein was gradually increased for 48 h after *SCRIB* knockdown ( Supplementary Figures S1 and S2).

In summary, the interacting proteins of FAP were screened by immunoprecipitation (IP), and the interaction between FAP and SCRIB was confirmed first by co-IP and a yeast two-hybrid system. The downregulation of SCRIB protein inhibits the apoptosis of SCC-9 cells and promotes cell growth, while the levels of FAP, p-P13K and p-Akt are increased, and the level of cleaved Caspase3 is decreased significantly. This indicates that *SCRIB* knockdown inhibits SCC-9 cell apoptosis via the PI3K/Akt signaling pathway by negatively regulating FAP expression. In future studies, the molecular mechanism by which SCRIB interacts with FAP and mediates downstream apoptotic factors (PI3K and Akt) to promote OSCC cell apoptosis will be investigated. The regulation of FAP on SCRIB protein level will be studied. The key amino acids, regions and sites of SCRIB that interact with FAP, ultimately leading to characteristic cell morphogenesis changes and apoptosis, will be determined. These studies will reveal the regulatory mechanism of the interaction between SCRIB and FAP in the occurrence and development of OSCC and provide new ideas for targeted therapy of OSCC.

## Supporting information

544FigS1-2-TabS1

## Supplementary Data

Supplementary data is available at *Acta Biochimica et Biphysica Sinica* online.
