# Supplementary material for: Identification and preliminary study of a novel interacting protein SCRIB with fibroblast activation protein in OSCC: SCRIB interacts with fibroblast activation protein in OSCC
Source: Acta Biochim Biophys Sin (Shanghai). 2023 Jun 19;55(8):1327–30. doi: 10.3724/abbs.2023102 (PMC10448038; doi:10.3724/abbs.2023102)
Supplement: 544FigS1-2-TabS1 [file 544FigS1-2-TabS1.pdf]

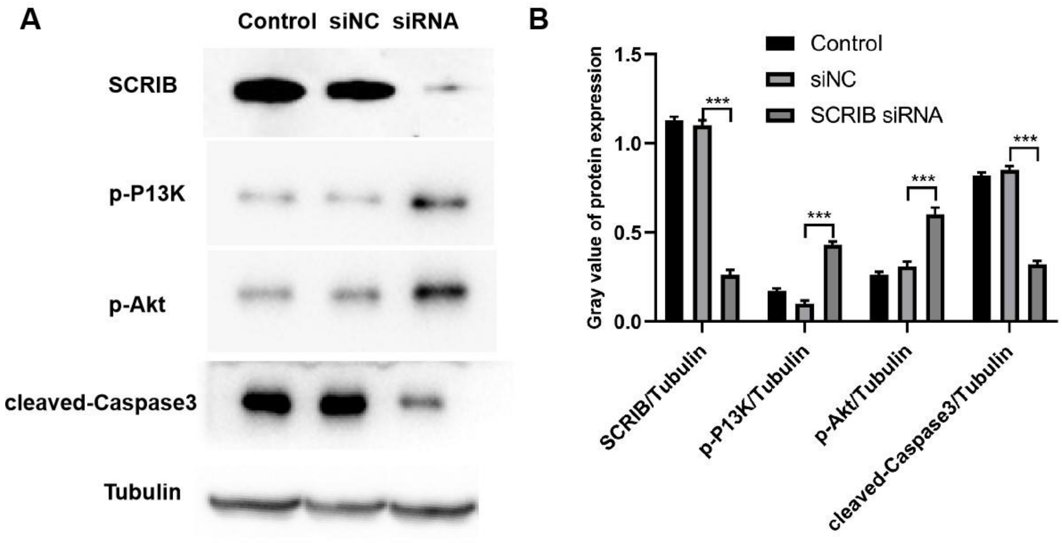

Supplementary Figure S1. The detection of p-P13K, p-Akt, cleaved Caspase3 and FAP protein levels after *SCRIB* knockdown (A) The levels of p-P13K, p-Akt and cleave-Caspase3 were detected by western blot analysis after *SCRIB* knockdown. (B) The levels of p-P13K, p-Akt and cleave-Caspase3 protein expression were analyzed.

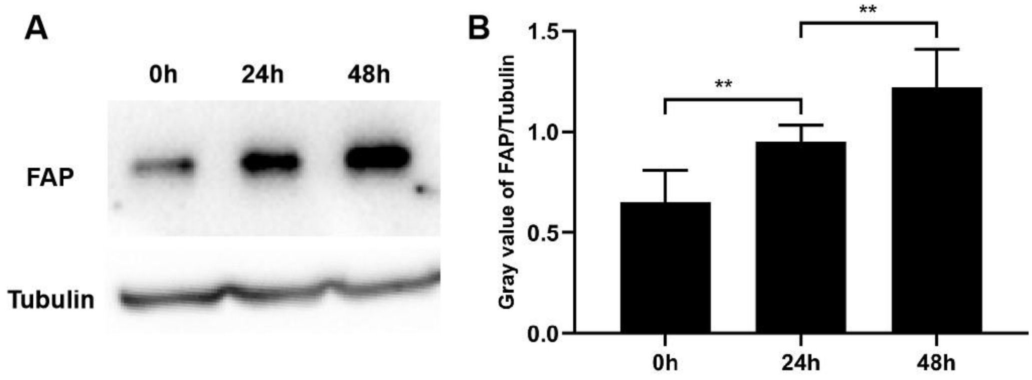

Supplementary Figure S2. The detection of FAP protein level after *SCRIB* knockdown (A) The levels of FAP protein was detected by western-blot analysis at 0 h, 24 h and 48 h after *SCRIB* knockdown. (B) The gray values of FAP protein expression were analyzed.

Supplementary Table S1. The sequences of RNAs used in this study

siRNA name	Sense (5′→3′)	Anti-sense (5′→3′)
SCRIB-siRNA 1	CCCUGUCAUUUCUGGUCAATT	UUGACCAGAAAUGACAGG GTT
SCRIB-siRNA 2	CCCUGAAUGAUGUGUCUCUTT	AGAGACACAUCAUUCAGG GTT
SCRIB-siRNA 3	GGAACGAUAUCCCUGAGAUTT	AUCUCAGGGAUAUCGUUC CTT
NC-siRNA 1	UUCUCCGAACGUGUCACGUTT	ACGUGACACGUUCGGAGA ATT
